# Supplementary material for: The Role of Petrimonas mucosa ING2-E5AT in Mesophilic Biogas Reactor Systems as Deduced from Multiomics Analyses
Source: Microorganisms. 2020 Dec 17;8(12):2024. doi: 10.3390/microorganisms8122024 (PMC7768429; doi:10.3390/microorganisms8122024)
Supplement: Supplementary file 1 [file microorganisms-08-02024-s001.zip › Supporting information/Supporting information.docx]

**Supporting information**

**Supporting Information Table 1:** List of publicly available metagenome datasets originating from the RefSeq used for fragment recruitment analysis. Provided as Excel file.

**Supporting information Table 2:** Characteristics and fed substrates of the 13 different biogas and wastewater treatment plants analyzed in the scope of the metatranscriptome analysis.

| **Biogas plant** | **Scale** | **Process Temperature** | **Fed substrate** | **Reference** |
| --- | --- | --- | --- | --- |
| BGP_1 | full scale | 40 °C | maize silage, dried poultry manure, cow manure | [^16^] |
| BGP_2 | full scale | 40 °C | maize silage, cow manure, chicken manure | [^92^] |
| BGP_3 | full scale | 54 °C | maize silage, pig slurry, cattle slurry, chicken dung, organic waste | [^93^] |
| BGP_4 | full scale | 52 °C | maize silage, grass silage | [^93^] |
| BGP_5 | full scale | 40 °C | grass silage, maize silage, grain, cattle slurry | [^93^] |
| WWTP_1 | full scale | - | pig farm waste water | [^60^] |
| WWTP_2 | 3 L | 55 °C | cheese whey permeate, cheese waste powder, hydrogen injection | [^94^] |
| BGP_6 | 10 L + 10 L | 37 °C | silage of perennial ryegrass | [^9^] |
| BGP_7 | 10 L + 10 L | 55 °C | silage of perennial ryegrass | [^9^] |
| BGP_8 | 17 L | 41 °C | maize silage and inoculated slurry from BGP_11 | [^85^] |
| BGP_9 | 17 L | 35 °C to 41 °C | maize silage and inoculated slurry from BGP_11 | [^85^] |
| BGP_10 | full scale | n.d | renewable energy crops and slurry from cows, pigs, chicken, sheep, horses | [^95^] |
| WWTP_3 | lab scale | n.d | n.e. | NCBI bioproject PRJEB20771 |

Abbreviations: BGP: Biogas plant; WWTP: Wastewater treatment plant; n.d.: not determined; n.e.: not existing.

**Supporting information Table 3:** Statistics of the obtained and processed metatranscriptome sequences analyzed in order to investigate the transcriptional profile of *P. mucosa*.

| **Dataset** | **Source of sample** | **Total number of reads** | **Sequences Assigned to *Petrimonas**** | **NCBI accessions number** | **Notes** |
| --- | --- | --- | --- | --- | --- |
| 1 | BGP_1 | 16,744,487 | 0.05% | SRR6214931 | sampling point at 82 d |
| 2 |  | 17,096,627 | 0.03% | SRR6214922 | sampling point at 187 d |
| 3 |  | 17,186,438 | > 0.01% | SRR6214916 | sampling point at 226 d |
| 4 |  | 16,028,864 | > 0.01% | SRR6214934 | sampling point at 253 d |
| 5 |  | 14,033,632 | 0.06% | SRR6214936 | sampling point at 553 d |
| 6 |  | 14,817,946 | 0.06% | SRR6214911 | sampling point at 587 d |
| 7 | BGP_2 | 82,269,139 | 0.06% | SRR2917899 | n.e. |
| 8 | BGP_3 | 13,670,038 | > 0.01% | ERR2672710 | n.e. |
| 9 | BGP_4 | 5,478,555 | 0.05% | ERR2672711 | n.e. |
| 10 | BGP_5 | 18,092,879 | 0.14% | ERR2672712 | n.e. |
| 11 | WWTP_1 | 42,084,918 | > 0.01% | SRR10972806, SRR10972807, SRR10972808 | n.e. |
| 12 | WWTP_2 | 5,613,031 | 0.07% | SRR6032681, SRR6032680, SRR6032651 | before hydrogen injection |
| 13 |  | 2,402,022 | 0.03% | SRR6032687, SRR6032686, SRR6032685 | after hydrogen injection |
| 14 |  | 11,418,619 | > 0.01% | SRR6032650, SRR6032608, SRR6032606 | before hydrogen injection |
| 15 |  | 9,539,618 | > 0.01 % | SRR6032685, SRR6032683, SRR6032682 | after hydrogen injection |
| 16 | BGP_6 | 45,754,958 | 0.17% | ERR3010911, ERR3010910, ERR3010909, ERR3010916 | low organic loading rate |
| 17 |  | 102,122,022 | 0.06% | ERR3010907, ERR3010903, ERR3010902, ERR3010901 | increased organic loading rate |
| 18 | BGP_7 | 35,003,029 | > 0.01% | ERR3010915, ERR3010914, ERR3010913, ERR3010912 | low organic loading rate |
| 19 |  | 125,683,154 | 0.07% | ERR3010908, ERR3010906, ERR3010905, ERR3010904 | increased organic loading rate |
| 20 | BGP_8 | 13,354,119 | 0.23% | ERR2029735 | sampling point: 7 d after inoculation |
| 21 |  | 11,431,267 | 0.38% | ERR2029737 | sampling point: 21 d after inoculation |
| 22 |  | 8,268,921 | 0.44% | ERR2029739 | sampling point: 42 d after inoculation |
| 23 |  | 9,785,248 | 0.41% | ERR2029740 | sampling point: 82 d after inoculation |
| 24 | BGP_9 | 12,142,259 | 0.23% | ERR2029736 | sampling point: 7 d after inoculation |
| 25 |  | 13,273,954 | 0.24% | ERR2029738 | sampling point: 21 d after inoculation |
| 26 |  | 8,756,172 | 0.25% | ERR2029740 | sampling point: 42 d after inoculation |
| 27 |  | 8,188,859 | 0.53% | ERR2029742 | sampling point: 82 d after inoculation |
| 28 | BGP_10 | 11,362,705 | 0.22% | ERR2029734 | n.e. |
| 29 | WWTP_3 | 43,492,450 | 0.09% | ERR1960624, ERR1960625, ERR1960626 | n.e. |

* The fourth column refers to the metatranscriptome reads that could be mapped to any metatranscriptome contig assigned to the species *Petrimonas*, as a fraction of all reads mapped to metatranscriptome contigs in the sample. Abbreviation: n.e. - not existing.

**Supporting Information Table 4:** *P. mucosa* ING2-E5A^T^ most actively transcribed genes for enzymes involved in carbohydrate or protein utilization in 29 analyzed datasets originating from different biogas plants. Provided as Excel file.
